# Supplementary material for: New Evidence of Significant Association between EBV Presence and Lymphoproliferative Disorders Susceptibility in Patients with Rheumatoid Arthritis: A Systematic Review with Meta-Analysis
Source: Viruses. 2022 Jan 10;14(1):115. doi: 10.3390/v14010115 (PMC8781518; doi:10.3390/v14010115)
Supplement: Supplementary file 1 [file viruses-14-00115-s001.zip › viruses-1521568-supplementary.pdf]

Supplementary material

Table S1. Systematic review

Author, year	Study design	n RA-LPD	n Controls	WHO classification of LPD	LPD stage	RA activity	Controls/cohort characteristics	Age Cases vs. Controls	Gender, M/F	Matching	EBV+ Cases	EBV+ Controls	Specimen for EBV detection	EBV target	Latency	EBV detection method
Kamel OW, 1994 [1] USA	NR	15 Total<br>1 DML<br>9 DLCL<br>1 MCHD<br>3 FLCL<br>1 FSCL	3 Total<br>2 LPI<br>1 NSHD	DML - diffuse mixed lymphoma, DLCL - diffuse large cell lymphoma, MCHD - mixed celularity, HL - Hodgkin`s disease, FLCL - follicular large-cell lymphoma, FSCL - follicular small-cleaved-cell lymphoma	NR	NR	patients with dermatomyositis and a diagnosis of lymphoma or lymphoproliferative lesion (LPI - lymphoplasmacytic infiltrate 2 patients, NSHD - nodular sclerosing Hogkin`s disease 1 patient)	67.9 (SD 13.3)<br>Vs.<br>40.6 (SD 25.5)	2/13<br>Vs.<br>3/3	NR	4	1 - LPI<br>1- NSHD	biopsy	anti LMP Ab<br>EBER1	NR	Immunohistochemistry<br>in situ hybridization
Bachman TR, 1996 [2] USA	case series	2	NR	Case 1 - Anaplastic large lymphoma with probable B phenotype<br>Case 2 - Diffuse large cell lymphoma with B cell phenotype (DLBCL)	NR	NR	NO CONTROL GROUP	65 years<br>66 years	1/1	NR	2	NR	biopsy	EBER1	NR	in situ hybridization
Van de Rijn M, 1996 [3] USA	NR	2	NR	BCL - B-cell lymphoma	NR	NR	NO CONTROL GROUP	NR	0/2	NR	2	NR	biopsy	EBER	NR	in situ hybridization
Georgescu L, 1997 [4] USA	case series	2	NR	NHL - non-Hodkin`s lymphoma	NR	NR	NO CONTROL GROUP	Case 1 - 54 years<br>Case 2 - 77 years	0/2	NR	0	NR	biopsy	NR	NR	Souther blot, PCR
Natkunam Y, 1997 [5] USA	NR	8 Total<br>2 LPD-HD<br>6 DLCL	2 Total<br>1 DM with HD<br>1 DM with ALPD	LPD-HD - Lymphoroliferative disorder resembling Hodgkin`s disease, DLCL - diffuse large cell lymphoma	NR	NR	Dermatomyositis patients who developed LPD (HD - Hodgkin`s disease 1 patient and ALPD - atypical lymphoproliferative disorder 1 patient)	NR	NR	NR	8 EBER1<br>7 lmp<br>8 EBNA-2	2 EBER1<br>2 LMP<br>2 EBNA -2	biopsy	EBER1<br>LMP and EBNA-2	NR	in situ hybridization<br>PCR

Kleiman KS, 1998 [6] USA	case series	4	NR	Case 1 - Diffuse large cell immunoblastic lymphoma<br>Case 2 - Hodgkin`s lymphoma Anaplastic large cell lymphoma<br>Case 3 - BCL - B-cell lymphoma<br>Case 4 - NHL - non-Hodgkin`s lymphoma	NR	RF titer<br>Case 1 - increased from 1/320 to 1/1310720<br>Case 2 - 1/2560; nephelometry from 132 U/mL to 38 U/mL<br>Case 3 - decreased from 331 U/mL, 243 U/mL to 38 U/mL<br>Case 4 - RF negative	NO CONTROL GROUP	Case 1 - 71 years<br>Case 2 - 78 years<br>Case 3 - 75 years<br>Case 4 - 68 years	3/1	NR	2	NR	biopsy	EBER	NR	in situ hybridization
Kojima M, 1998 [7] Japan	NR	5	18	PTLD - Florid follicular hyperplasia	NR	NR	P tients florid reactive follicular hyperplasia without RA	72.4 (SD 6.2)<br>Vs.<br>66.6 (SD 4.6)	2/3<br>Vs.<br>8/10	NR	1 EBER	6 EBER	biopsy	EBERs	NR	in situ hybridization
Chevrel G, 1999 [8] France	case series	2	NR	Case 1 and Case 2 - Polymorphic diffuse B cell lymphoproliferation	NR	NR	NO CONTROL GROUP	Case 1 - 64 years<br>Case 2 - 67 years	1/1	NR	1	NR	biopsy	EBERs	NR	in situ hybridization
Goodlad JR, 1999 [9] Multicenter (UK, USA)	case series	3	NR	Case 1, 2, and 3 - NHL - non-Hodgkin`s lymphoma	NR	NR	NO CONTROL GROUP	Case 1 - 33 years<br>Case 2 - 67 years<br>Case 3 - 76 years	0/3	NR	0	NR	biopsy	EBER1 EBER2	NR	in situ hybridization
Menke DM, 2000 [10] USA	NR	23 Total<br>3 HL<br>10 NHL<br>1 SLL<br>1 LPL<br>5 LBCL<br>2 BL<br>1 FL	21 Total<br>1 HL<br>10 NHL<br>1 LPL<br>2 MALT<br>5 LBCL<br>1 BL<br>1 FL	HL - Hodgkin`s lymphoma, NHL - non-Hodgkin`s lymphoma, SLL - small lymphocytic lymphoma, LPL - lymphoplasmocitoid lymphoma, LBCL - large B-cell lymphoma, BLL - Burkitt-like lymphoma, FL - follicular lymphoma	NR	NR	RA-LPD without MTX	NR	5/8	NR	3/3 HL<br>4/10 NHL<br>0/1 SLL<br>0/1 LPL<br>2/5 LBCL<br>1/2 BL<br>1/1 FL	1 /1HL<br>1/10 NHL<br>0/1 LPL<br>0/2 MALT<br>1/5 LBCL<br>0/1 BL<br>0/1 FL	biopsy	EBERs<br>anti LMP Ab<br>BamH1W	NR	in situ hybridization<br>immunohistochemistry<br>PCR
Dawson TM, 2001 [11] USA	NR	19	6	Large cell lymphoma, Small cell lymphoma, HL - Hodgkin`s lymphoma, FL - follicular lymphoma, Mixed cell lymphoma	NR	NR	Sjogren syndrome LPD patients	Median 62 (range 40-81)<br>Vs.<br>Median 69 (range 58-77)	6/13<br>Vs.<br>1/5	NR	5/19	1/6	biopsy	EBER	NR	in situ hybridization

Tournadre A, 2001 [12] France	NR	1	1	TCL - T-cell lymphoma	NR	NR	dermatomyositis LPD patient	Individual data 70 Vs. 47	1/0 Vs. 0/1	NR	1	1	biopsy	EBER	NR	in situ hybridization
Mariette X, 2002 [13] France	prospective	25	NR	HL - Hodgkin`s lymphoma NHL - non-Hodgkin`s lymphoma	NR	NR	NO CONTROL GROUP	Mean 63 (range 39-82)	10/15	NR	8/25	NR	biopsy	EBERs anti LMP Ab EBNA1	NR	in situ hybridization immunohistochemistry PCR
Baecklund E, 2003 [14] Sweden	case-control	35 Total 2 HL 33 NHL: 22 DLBCL 1 hepatosplenic T cell lymphoma 2 FL 1 lymphoplasmacytic lymphoma 1 Burkitt`s lymphoma 2 unspecified high-grade NHL 1 unspecified low-gfrade NHL 1 unspecified NHL	NR	HL - Hodgkin`s lymphoma, NHL - non-Hodgkin`s lymphoma: DLBCL - diffuse large B cell lymphoma, hepatosplenic T cell lymphoma, FL - follicular lymphoma, lymphoplasmocytic lymphoma, BL - Burkitt`s lymphoma, unspecified high-grade NHL, unspecified low-grade NHL, and unspecified NHL	NR	ESR, number of tender and swollen joints, and physician`s global assessment of disease activity: Low (ESR 1-30, joints 0-3) 2 patients Medium (ESR 31-60, joints 4-6) 14 patients High (ESR≥61, joints≥7) 19 patients	NO CONTROL GROUP	NR	NR	NR	0/1 HL 4/22 DLBCL 0/1 hepatosplenic T cell lymphoma 0/2 FL 1/1 lymphoplasmacytic lymphoma 0/1 Burkitt`s lymphoma 0/2 unspecified high-grade NHL 0/1 unspecified low-gfrade NHL 0/1 unspecified NHL	NR	biopsy	EBER	NR	in situ hybridization
Magro CM, 2004 [15] USA	retrospective	2	9	plasmacytic marginal zone lymphoma with reactive germinal centers and a background population of small marginal zone neoplastic B lymphocytes	NR	NR	T cell rich plasmacytic marginal zone lymphoma and plasmacytic marginal zone lymphoma unaccompanied by a significant small lymphocytic infiltrate without RA	71.5 (SD 2.12) Vs. 66.0 (SD 1.41)	0/2 Vs. 6/3		0/2	1/9	biopsy	EBER	NR	in situ hybridization
Verma S, 2005 [16] USA	NR	1	5	DLBCL - Difuse large B-cell lymphoma	NR	NR	posttransplant patients with LPD	75 Vs. 39.8±7.79	0/1 Vs. 3/2	NR	1	5	biopsy	EBER	NR	in situ hybridization

Au WY, 2006 [17] China	NR	2	9	BCL - B-cell lymphoma	NR	NR	Patients with other autoimmune diseases (SLE, UC - ulcerative colitis, psoriasis, CFA-cryptogenic fibrosing alveolitis, VKH - Vogts-Kaganagi-Harada syndrome) and lymphoma	63.5 (SD 4.9) Vs. 49.0 (SD 9.9)	0/2 Vs. 4/5	NR	2	3	biopsy	EBERs	NR	in situ hybridization
Baecklund E, 2006 [18] Sweden	case-control	378 Total<br>269 BCL<br>16 TCL and NK cell neoplasms<br>21 HL<br>37 Unspecified lymphomas	378	BCL<br>TCL and NK cell neoplasms<br>HL<br>Unspecified lymphoma	I, II, III, IV	- ESR, number of tender and swollen joints, and physician`s global assessment of disease activity the overall disease activity was: Low (ESR 1-30, joints 0-3) 94/278 cases/controls; Medium (ESR 31-60, joints 4-6) 196/94 cases/controls; High (ESR≥61, joints≥7) 86/4 cases/controls<br>- cumulative disease activity, as the duration (in months) of 4 different levels:<br>I - inactive 34/138 cases/controls<br>II - low 185/204 cases/controls<br>III - medium 105/31 cases/controls<br>IV - high 52/3 cases/controls	Lymphoma-free individuals from the RA cohort without any registered lymphoma at the time lymphoma was diagnosed in the corresponding RA case. Controls were individually matched according to sex, year of birth, year of first hospital discharge with a diagnosis of RA, and country of residence.	NR	170/208 Vs. 170/208	Yes (sex, year of birth, year of first RA discharge, and county of residence)	37/304 Total<br>23/258 BCL<br>2/15 TCL and NK cell neoplasms<br>9/19 HL<br>3/12 unspecified lymphoma	NR	biopsy	EBER	NR	in situ hybridization

Hoshida Y, 2006 [19] Japan	NR	76 Total<br>60 BCL (44 DLBCL, 3 diffuse polymorphic, 1 HL-like LPD, 12 other)<br>7 TCL<br>1 NK cell lymphoma 8 HL<br>48 MTX treated<br>28 non-MTX treated	150 Total<br>111 BCL (64 DLBCL, 23 FL, 4 lymphoplasmocytic, 1 plasmocytoma, 3 mantle cell lymphoma, 16 others) 16 TCL<br>6 NK/T cell LPD<br>16 HL	BCL (DLBCL, FL, lymphoplasmocytic, plasmocytoma, mantle cell lymphoma, diffuse plymorphic, HL-like LPD, other), TCL, NK cell lymphoma, HL	I, II, III, IV; undetermined	NR	Sporadic LPD patients (BCL - DLBCL, FL, lymphoplasmocytic, plasmocytoma, mantle cell lymphoma, other; TCL; NK/T cell LPD; HL)	Median 66 (range 23-87)<br>Vs.<br>Median 58 (range 17-87)	21/55<br>Vs.<br>93/57	NR	10/60 BCL (6/44 DLBCL, 3/3 diffuse polymorphic, 1/1 HL-like LPD, 0/12 other)<br>3/7 TCL<br>1/1 NK cell lymphoma<br>7/8 HL<br>13/48 MTX treated<br>8/28 non-MTX treated	2/87 BCL (2/50 DLBCL, 0/0 diffuse polymorphic, 0/0 HL-like LPD, 0/37 others)<br>2/9 TCL<br>4/5 NK/T cell LPD<br>3/8 HL	biopsy	EBER	NR	in situ hybridization
Kojima M, 2006 [20] Japan	case-report	13 Total<br>4 DLBCL<br>3 HL<br>2 MALT<br>1 peripheral T-cell lymphoma<br>3 PSLLPI	NR	DLBCL - diffuse large B-cell lymphomas, HL - Hodgkin`s lymphoma, MALT - marginal zone B-cell lymphoma, peripheral T-cell lymphoma, PSLLPI - polymorphus, small lymphocytic or lympoplasmacytic infiltrate	IA, IB, IEA, IAE,<br>IIIB, IVAE, IVBE	NR	NO CONTROL GROUP	Median 63 (range 52-86)	4/9	NR	7/13 EBER<br>4/11 LMP	NR	biopsy	EBER<br>LMP	NR	in situ hybridization<br>immunohistochemistry
Kojima M, 2006 [21] Japan	case-report	17 Total<br>3 HL<br>18 BCL<br>3 TCL<br>4 HL<br>2 MALT<br>12 DLBCL<br>2 angioimmunoblastic T-cell lymphoma<br>1 primary cutaneous T-cell lymphoma	7 Total<br>3 SLE<br>2 DM<br>2 SS	HL - Hodgkin`s lymphoma, BCL - B-cell lymphoma, TCL - T-cell lymphoma, FL - follicular lymphoma, MALT - marginal zone B-cell lymphoma, DLBCL - diffuse large B-cell lymphoma, Angioimmunoblastic T-cell lymphoma, Primary cutaneous T-cell lymphoma	I, II, III, IV	NR	Patients with Systemic Rheumatic Diseases other than RA, (SLE - systemic lupus erythematosus, DM - dermatomyositis, SS - systemic sclerosis)	NR	NR	NR	17 Total RA<br>3/3 HL<br>0/18 BCL<br>0/3 TCL<br>0/4 HL<br>0/2 MALT<br>0/12 DLBCL<br>0/2 angioimmunoblastic T-cell lymphoma<br>0/1 primary cutaneous T-cell lymphoma	7 Total<br>0/3 SLE<br>0/2 DM<br>0/2 SS	biopsy	EBER<br>LMP	NR	in situ hybridization<br>immunohistochemistry

Xu JX, 2007 [19] Japan	NR	53 Total<br>41 BCL (24 TTX + 17 non-MTX)<br>31 DLBCL (18 MTX + 13 non-MTX)<br>2 polymorphic (1 MTX + 1 non-MTX)<br>1 Hodgkin-like (1MTX)<br>6 TCL (2 MTX + 4 non-MTX)<br>5 HL (5 MTX)	NR	BCL - B-cell lymphoma<br>DLBCL - diffuse large B-cell lymphoma<br>polymorphic lymphoma<br>Hodgkin-like lymphoma<br>TCL - T-cell lymphoma<br>HL - Hodgkin`s lymphoma	I, II, III, IV	NR	NO CONTROL GROUP	MTX – median 67 (range 34-87)<br>Non-MTX - median 66 (range 23-77)	13/31	NR	14/53 Total<br>0/41 BCL (0/24 TTX + 0/17 non-MTX)<br>3/31 DLBCL (2/18 MTX + 1/13 non-MTX)<br>2/2 polymorphic (1/1 MTX + 1/1 non-MTX)<br>1/11 Hodgkin-like (1/1MTX)<br>4/6 TCL (1/2 MTX + 3/4 non-MTX)<br>4/5 HL (4/5 MTX)	NR	biopsy	EBER	NR	in situ hybridization
Miyazaki T, 2007 [22] Japan	retrospective	9 Total<br>8 DLBCL<br>1 PTCL-u	NR	DLBCL - diffuse large B-cell lymphoma, PTCL-u - peripheral T-cell lymphoma, unspecified	I, II, III, IV	CRP<br>1.09 (0.10-5.83) median (min-max)	NO CONTROL GROUP	Mean 65.0 (SD 7.8)	3/6	NR	3/9 Total<br>3/8 DLBCL<br>0/1 PTCL-u	NR	biopsy	EBER<br>LMP and EBNA2	2 latency III a 1 latency II	in situ hybridization immunohistochemistry
Kleinschmidt-DeMasters BK, 2008 [23] USA	NR	1	3	Polymorphus lymphoplasmacytic EBV associated LPD	NR	NR	Patients with neuropathy, polymiositis, myastenia gravis	Individual data 78<br>Vs.<br>65, 65, and 80 years	0/1<br>Vs.<br>2/1	NR	1	3	biopsy	EBER	NR	in situ hybridization
Hasserjian RP, 2009 [24] Multicenter (USA, Belgium)	NR	5	6	TCL - T-cell lymphoma, ALCL - anaplastic large cell lymphoma, FL - follicular lymphoma, Extranodal marginal zone B-cell lymphoma, HL - Hodgkin`s lymphoma	NR	NR	LPD (FL - follicular lymphoma, DLBCL - diffuse large B-cell lymphoma, HL - Hodgkin`s lymphoma, LPL - lymphoplasmacytic lymphoma) patients with psoriasis or Chron`s disease	59.8 (SD 11.3)<br>Vs.<br>61.6 (SD 12.5)	0/5<br>Vs.<br>3/3	NR	1/5	5/6	biopsy	EBER	NR	in situ hybridization

Hellgren K, 2010 [25] Sweden	cohort	378	378	DLBCL - diffuse large B-cell lymphoma, FL -follicular lymphoma, Other BCl, T/NK cell lymphoma, HL - Hodgkin`s lymphoma, Unspecified lymphomas, EBV+ lymphomas	I, II, III, IV	-ESR, number of tender and swollen joints, and physician`s global assessment of disease activity the overall disease activity was: Inactive, Low, Medium, and High -Cumulative disease activity for the whole RA period was calculated as the area under the curve (AUC) for the duration in months of the four different levels of disease activity.	From the RA cohort (n=74 651) three patients were randomly selected as potential controls, matched for sex, year of birth, year of first RA discharge and county of residence. Controls had to be alive and without any registered cancer at the time of the lymphoma diagnosis of their corresponding case. Among these potential controls we included the first of the three controls whose medical record could be identified and who fulfilled the American College of Rheumatology criteria for RA	NR	170/208<br>Vs.<br>170/208	Yes (sex, year of birth, year of first RA discharge, and county of residence)	32/343	NR	biopsy	EBER	NR	in situ hybridization
Mariette X, 2010 [26] France	NR	27 Total<br>22 non-HL<br>20 BCL<br>11 DLBCL<br>3 FL<br>1 marginal zone B-cell lymphoma<br>1 lymphocytic lymphoma<br>4 other<br>2 TCL<br>2 pleimorphic T-cell lymphoma<br>5 HL and Hodgkin`s like lymphoma	54	non-HL - non-Hodgkin`s lymphoma, BCL - B-cell lymphoma, DLBCL - diffuse large B-cell lymphoma, FL - follicular B-cell lymphoma, Marginal zone B-cell lymphoma, Lymphocytic B cell lymphoma, Other B-cell lymphomas, TCL - T-cell lymphoma, Pleimorphic TCL, HL - Hodgkin`s lymphoma, Hodgkin`s like lymphoma	NR	NR	Lymphoma-free patients receiving anti-TNF treatment in a labelling indication were included from centres participating in the RATIO registry (thus from the same population source) in a global pool of controls. From that pool, we randomly selected patients for a database of controls reflecting the proportion of patients receiving each of the three anti-TNF drugs in France. Two controls per case were randomly matched by sex, age (within 5 years) and underlying inflammatory disease from this database of controls. We also used a second sample of controls randomly selected from the same database of controls, with the same matching criteria (second matching).	63.4 (SD 11.8)	12/15	Yes (sex, age (within 5 years), and underlying inflammatory disease)	2/27 Total<br>0/22 non-HL<br>0/20 BCL<br>0/11 DLBCL<br>0/3 FL<br>0/1 marginal zone B-cell lymphoma<br>0/1 lymphocytic lymphoma<br>0/4 other<br>0/2 TCL<br>0/2 pleimorphic T-cell lymphoma<br>2/5 HL and Hodgkin`s like lymphoma		biopsy	EBER	NR	in situ hybridization
Kojima M, 2010 [27] Japan	NR	3	6	ALPIB - atypical lymphoplasmacytic immunoblastic proliferation (rare LPD)	NR	All 3 RA cases had active form	Rheumatic diseases other than RA (SLE - systemic lupus erythematosus, DM -dermatomyositis, SJS - Sjogren`s syndrome)	62.3 (SD 12.5)<br>Vs.<br>42.3 (SD 13.5)	0/3<br>Vs.<br>0/6	NR	0/3	0/6	biopsy	EBER	NR	in situ hybridization

Nakamichi N, 2010 [28] Japan	NR	6	13	polymorphus LPD – with neither diagnostic Reed-Sternberg (RS) cells nor a substantial number of medium-to-large T cells with nuclear hyperchromasia, and variable numbers of bizarre cells with multilobed nuclei, indicating that diagnoses of HL or PTCL were unlikely in these cases. Cases with monoclonal rearrangement of the TCR gene, but without specific characteristics, such as positive anti-human T lymphotropic virus type I (HTLV-I) antibody and/or monoclonal integration of HTLV-1 proviral DNA in the tumor cells, and appearance of clusters of “clear cells,” were classified as PTCL-NOS. Cases showing diffuse proliferation of large B-lymphoid cells with a prominent polymorphous cellular background containing small lymphoid cells, plasma cells, histiocytes, and/or eosinophils that appeared neoplastic were classified as DLBCL-NOS. Cases in individuals >50 years, with the EBV genome in the nuclei of proliferating cells, with no known immunodeficiency or prior lymphoma, were classified as EBV+ DLBCL-E. LPD that arose in patients treated with immunosuppressive drugs for autoimmune diseases or conditions other than an allograft/ autograft transplant setting were classified as other iatrogenic immunodeficiency-associated LPD	I, II, III, IV	Performance status (PS) PS 0 - 2 patients PS 1 - 2 patients PS 4 - 2 patients	Patients with polymorphus LPD without immune abnormalities and without RA	68.7 (SD 10.0) Vs. 54.5 (SD 18.4)	0/6 Vs. 7/6	NR	4/6	4/13	biopsy	EBER	NR	in situ hybridization
Niitsu N, N, 2010 [29] Japan	NR	29	NR	DLBCL - diffuse large B-cell lymphoma	I, II, III, IV	Performance status (PS) PS 0,1 - 25 patients PS 2-4 - 4 patients	NO CONTROL GROUP	Median 62 (range 43-78)	8/21	NR	7/29	NR	biopsy	EBER	NR	in situ hybridization

Löfström B, 2011 [30] Sweden	NR	95	12 SLE<br>63 Comparator cases	DLBCL - diffus large B-cell lymphoma	I, II, III, IV	-ESR, number of tender and swollen joints, and physician`s global assessment of disease activity the overall disease activity was: Inactive, Low, Medium, and High<br>-Cumulative disease activity for the whole RA period was calculated as the area under the curve (AUC) for the duration in months of the four different levels of disease activity.	SLE-LPD (DLBCL)<br>Comparator cases were DLBCL patients without chronic inflammatory disease (EBV not assessed)	50.8 (SD 13.7)<br>Vs.<br>SLE 45.8 (SD 14.6)	42/53<br>Vs.<br>32/43	NR	10/91	1/8 SLE	biopsy	EBER	NR	in situ hybridization
Minamimoto R, 2011 [31] Japan	NR	5	1	DLBCL - diffuse large B-cell lymphoma, HL - Hodgkin`s lymphoma, peripheral T-cell lymphoma	NR	NR	Psoriasis with LPD (DLBCL)	67 (SD 10.0)<br>Vs.<br>42 years	1/4<br>Vs.<br>1/0	NR	5/5	NR	biopsy	anti-EBV CA Ab	NR	ELISA
Tokuhira M, 2012 [32] Japan	NR	23	NR	DLBCL - diffuse large B-cell lymphoma, DLBCL-LYG - diffuse large B-cell lymphoma-lymphogranulomatosis, FL - follicular lymphoma, PTCL-NOS - peripheral T-cell lymphoma-not otherwisw specified, MALT - mucosal associated lymphoid tissue, MDLBCL - mediastinal diffuse large B-cell lymphoma, HL - Hodgkin`s lymphoma, HL-like - Hodgkin`s like lymphoma, Unclassified LPD	I, II, III, IV	CRP<br>5.3 (0.1-32.3) median (min-max)	NO CONTROL GROUP	Mean 62.7	7/16	NR	7/23	NR	biopsy	EBER	NR	in situ hybridization

Ichikawa A, 2013 [33] Japan	NR	102	NR	DLBCL - diffuse large B-cell lymphoma, HL - Hodgkin`s lymphoma, Polymorphic B-cell LPD, FL - follicular lymphoma, PTCL - peripheral T-cell lymphoma, Reactive lymphoadenitis, Composite lymphoma	I, II, III, IV	NR	NO CONTROL GROUP	Median 69 (range 42-88)	18/84	NR	TOTAL 56/93<br>23/49 DLBCL<br>3/8 HL<br>14/19 Polymorphic B-cell LPD<br>1/3 FL<br>3/3 PTCL<br>5/9 Reactive lymphoadenitis<br>2/2 Composite lymphoma	NR	biopsy	EBER<br>LMP and EBNA2	NR	in situ hybridization immunohistochemistry
Kondo S, 2013 [34] Japan	NR	54	NR	Mature B LPD, DLBCL - diffuse large B-cell lymphoma, Myeloma, MALT - mucosal associated lymphoid tissue, MCL - Mantle cell LPD, Mature T/NK cell LPD, HL - Hodgkin`s lymphoma	I, II, III, IV	NR	NO CONTROL GROUP	MTX median 66 (range 55-75) non-MTX median 65 (range 41-77)	13/41	NR	TOTAL 13/28 MTX treated<br>2/26-Non-MTX<br>Mature B LPD (6/15MTX I 1/22 non-MTX)<br>DLBCL (6/14 MTX and 1/16 non-MTX)<br>Myeloma (0/0 MTX and 0/1 non-MTX)<br>MALT (0/0MTX and 0/5 non-MTX)<br>MCL (0/1 MTX and 0/0 non-MTX)<br>Mature T/NK cell LPD (4/8 MTX and 0/2 non-MTX)<br>HL (3/5 MTX and 1/2 non-MTX)	NR	biopsy	EBER<br>LMP and EBNA2	NR	in situ hybridization immunohistochemistry
Loo EY, 2013 [35] USA	NR	5	5	Classical Hodgkin`s lymphoma	I, II, III, IV	NR	Patients with autoimmune diseases (DM - dermatomyositis, autoimmune hepatitis, Waldenstrom macroglobulinemia, Crohn disease, SLE - systemic lupus erythematosus)	58.8 (SD 17.2) Vs. 45.8 (SD 12.0)	1/4 Vs. 0/5	NR	3/5 EBER<br>1/4 LMP	5/5 EBER 1/5 LMP	biopsy	EBER<br>LMP	I 3/8<br>II 5/8	in situ hybridization immunohistochemistry

Yamakawa N, 2014 [36] Japan	NR	20	1	p-LPD - polymorphic lymphoproliferative disorder, HL - Hodgkin`s lymphoma, SBL - small B cell lymphoma, DLBCL - diffuse large B cell lymphoma	I, II, III, IV	Steinbrocker's classification: stage I - 1 patient stage II - 3 patients stage III - 3 patients stage IV - 13 patients	PM - polymyosistis with LPD	66.3 (SD 7.3) Vs. 53 years	4/16 Vs. 0/1	NR	11/19 Total pLPD 6/6 HL 2/3 DLBCL 2/9 SBL 1/1	1/1	biopsy	EBER LMP1 and EBNA2	I - 3/11 II - 5/11 III - 3/11	in situ hybridization immunohistochemistry
Kameda T, 2014 [37] Japan	nested case-control	28	125	DLBCL - diffuse large B cell lymphoma, HL - Hodgkin`s lymphoma, MCL - mantle cell lymphoma, Unknown	I, II	DAS28-CRP score at LPD onset was 2.26 (interquartile range 2.09–2.82) Steinbrocker's classification: stage I - 1 LPD, 13 non-LPD patients, stage II - 7 LPD, 53 non-LPD patients, stage III - 10 LPD, 21 non-LPD patients, stage IV - 6 LPD, 22 non-LPD patients	RA patients treated with MTX who didn`t develop LPD matched for age and sex	Mean 69.5 (range 52-92) Vs. Median 69.0 (range 63-75)	9/19	Yes (age and sex)	7/12 Total 16/28 not tested		biopsy and peripheral blood	EBER, EBV DNA viral load	NR	in situ hybridization, real-time PCR
Koens L, 2014 [38] Netherlands	NR	6	4	BCL - B cell lymphoma	NR	NR	patients treated with MTX with DM - dermatomyositis, PA - psoriatic arthritis, MS - morbus still)	77.7 (SD 11.5) Vs. 68.7 (SD 8.6)	3/3 Vs. 1/3	NR	2/6	3/4	biopsy	EBER, LMP1	NR	in situ hybridization, immunohistochemistry
Berghen N, 2015 [39] Belgium	prospective cohort	8	361 Total 26 with solid malignancy 335 without malignancy	NHL - non-Hodgkin`s lymphoma, HL - Hodgkin`s lymphoma, p-LPD - polymorphic lymphoproliferative disorder, MM - multiple myeloma, pre-T acute lymphocytic leukemia	NR	DAS-28 score - mean 5.18 (median 5.10)	RA patients with solid malignancy treted with anti-TNF therapy RA patients without malignancy treated with anti-TNF therapy	Mean 59 (range 38-72) Vs. Solid malignancy - Mean 58.7 No malignancy - Mean 51.5 (range 19.9-80.6)	NR	Yes (age)	3/4	NR	biopsy	EBV DNA in blood?	NR	real-time PCR
Hashimoto K, 2015 [40] Japan	case-series	2	NR	Both cases - Polymorphus B-cell LPD	NR	NR	NO CONTROL GROUP	Case 1 - 74 years Case 2 - 74 years	0/2	NR	2/2 polymorhous B-cell LPD	NR	peripheral blood	EBER LMP1 and anti-EBV Abs EBV DNA viral load	NR	in situ hybridization immunohistochemistry real-time PCR

Hollander P, 2015 [41] Denmark	case-control	585 (data for EBV were available for 498)	3187	HL - Hodgkin`s lymphoma	NR	NR	Controls were randomly sampled from the entire Danish and Swedish populations using continuously updated computerized population registers. They were frequency matched to controls within each country on the expected age (in 10-year strata) and sex distributions of the combined group of HL and NHL patients	age ranges: 18-34 years 277 (47.3%) 35-54 years 171 (29.2%) 55-74 years 137 (23.4%) vs. 18-34 312 (9.8%) 35-54 861 (27.0%) 55-74 2014 (63.2%)	305/280 Vs. 1767/1420	Yes (age and sex)	142/487 HL 8/21 RA and HL cases	NR	biopsy	EBER, LMP1	NR	in situ hybridization, immunohistochemistry
Inui Y, 2015 [42] Japan	retrospective	20 Total 13 BCL 2 TCL 5 HL	NR	BCL - B cell lymphoma, TCL - T cell lymphoma, HL - Hodgkin`s lymphoma	I, II, III, IV	NR	NO CONTROL GROUP	Median 70 (range 53-77)	5/15	NR	5/20 Total	NR	biopsy	EBER	NR	in situ hybridization
Shimizu S, 2015 [43] Japan	NR	1	1	DLBCL - Diffuse large B-cell lymphoma	NR	NR	dermatomyositis and Sjögren's syndrome LPD patient	Individual data 74 Vs 63 years	1/0 Vs. 0/1	NR	1	1	biopsy	EBER	NR	in situ hybridization
Yamada K, 2015 [44] Japan	NR	61 Total 42 BCL 40 DLBCL 1 FL 1 MCL 7 HL 12 T/NK cell lymphoma	35 Total 31 BCL 24 DLBCL 6 MZL 1 lymphoplasmacytic lymphoma 2 HL 2 T/NK cell lymphoma	BCL - B cell lymphoma, DLBCL - diffuse large B cell lymphoma, FL - follicular lymphoma, MCL - mantle cell lymphoma, HL - Hodgkin`s or Hogkin like lymphoma, T/NK cell type lymphoma	I, II, III, IV	NR	RA-LPD patients not treated with MTX (BCL - B cell lymphoma, DLBCL - diffuse large B cell lymphoma, MZL - marginal zone lymphoma, Lymphoplasmacytic lymphoma, HL - Hodgkin`s or Hogkin like lymphoma, and T/NK cell type lymphoma)	Median 65 (range 53-84) Vs. Median 67 (range 41-88)	12/49 Vs. 7/28	NR	25/40 MTX-BLPD	2/24 non-MTX BLPD	biopsy	EBER, LMP1, EBNA2	I - 2/25 MTX-BLDP 18/25 MTX-BLDP III - 5/25 MTX-BLDP II - 2/2 non-MTX BLDP lytic infection 7/25 MTX-BLDP	in situ hybridization, immunohistochemistry, ZEBRA-positive tumor cells (for lytic infection)
Jeon YW, 2016 [45] Korea	NR	4	7	NHL - non-Hodgkin`s lymphoma, DLBCL - diffuse large B cell lymphoma, MALT lymphoma, extranodal T/NK cell lymphoma	NR	CRP 0.79 (0.41-2.96) median (min-max)	non-RA (SS - Sjogren syndrome, Behcet disease, AS - ankylosing spondylitis, SLE - systemic lupus erythematosus), related non-Hodgkin`s lymphoma patients (DLBCL - diffuse large B cell lymphoma, MALT lymphoma, FL - follicular lymphoma, plasmablastic lymphoma)	65.5 (SD 5.9) Vs. 49.9 (SD 11.2)	2/2 Vs. 3/4		1/4	1/7	biopsy	EBER	NR	in situ hybridization

Watanabe S, 2016 [46] Japan	retrospective	13	2	FL - follicular lymphoma, DLBCL - diffuse large B cell lymphoma, MALT lymphoma, polymorphic BLPD, MZL - marginal zone lymphoma	I, II, III, IV	NR	PN (polyarteritis nodosa) and PA (psoriatic arthritis) patienst, with LPD (DLBCL) treated with MTX	Range 40-70 Vs. 50-60	NR	NR	5/13	2/2	biopsy	EBER	NR	in situ hybridization
Carroll M, 2017 [47] Australia	case-series	2	NR	Case 1 - Extranodal NK/T-cell lymphoma Case 2 - Plasmablastic lymphoma	NR	NR	NO CONTROL GROUP	Case 1 - 80 years Case 2 - 79 years	1/1	NR	1	NR	biopsy	EBER	NR	in situ hybridization
Ejima-Yamada K, 2017 [48] Japan	NR	49	22 Total 10 EBV+ DLBCL who had no history of RA, HIV infection, or transplants 12 RA patients with reactive lymphadenitis	B cell LPDs, DLBCL - diffuse large B cell lymphoma	I, II, III, IV	NR	Age- and sex-matched control patients with DLBCL (EBV– DLBCL non-germinal center B cell phenotype, EBV+ DLBCL) who had no history of RA, HIV infection, or transplants, and RA patients with reactive lymphadenitis	RA-LPD MTX median 68 (range 50-84) EBV+ RA-LPD MTX median 68 (range 56-82) EBV- RA-LPD MTX median 66 (range 50-84) RA-LPD non-MTX median 65 (range 41-88) Vs. Median 64 (range 38-90)	8/41 Vs. 6/15	Yes (age, sex)	21/38 RA related MTX-BLPD	15/44 (22 with EBV-DLBCL 10 EBV+ DLBCL who had no history of RA, HIV infection, or transplant 12 RA patients with related lymphadenitis	biopsy	EBER, LMP1, EBNA2	I - 5/12 RA patients with related lymphadenitis II -17/21 RA-related MTX-BLPD III-4/21RA-related MTX-BLPD	in situ hybridization, immunohistochemistry
Katsuyama T, 2017 [49] Japan	retrospective	30 Total 16 DLBCL 2 AITL 2 MZBCL 1 HL 2 MALT 1 NK/T cell 1 PTCL-NOS 3 other	NR	DLBCL - diffuse large B cell lymphoma, AITL - angioimmunoblastic T-cell lymphoma, MZBL - marginal zone B-cell lymphoma, MALT - mucosa associated lymphoid tissue, HL - Hodgkin`s lymphoma, PTCL-NOS - peripheral T-cell lymphoma not otherwise specified, other	NR	CRP 3.49 (SD 5.70)	NO CONTROL GROUP	68.2 (SD 9.7)	5/25	NR	9/12 EBV DNA 17/23 EBER	NR	biopsy and peripheral blood	EBER; EBV DNA	NR	in situ hybridization; PCR
Gion Y, 2017 [50] Japan	NR	51 Total 34 DLBCL 17 HL	NR	DLBCL - diffuse large B cell lymphoma, HL - Hodgkin`s lymphoma	I, II, III, IV	NR	NO CONTROL GROUP	Median 67 (range 45-84)	14/37	NR	28/34 DLBCL EBER HL: 14/17 EBER, 0/11 EBNA2, 8/13 lmp1	NR	biopsy	EBER; EBNA2; LMP1	I – 2 II – 6 III - 0	in situ hybridization; immunohistochemistry
Takanashi S, 2017 [51] Japan	cross-sectional	26	NR	BCL - B-cell lymphoma, TCL - T-cell lymphoma, HL - Hodgkin`s lymphoma, Reactive hyperplasia	I, II, III, IV	ECOG PS 0-1 77% 2-4 23% CRP 2.5 (0.1-28)	NO CONTROL GROUP	Median 67 (range 30-91)	3/23	NR	7/9	NR	biopsy	NR	NR	MEDICAL RECORD/HISTORY

Mariette X, 2018 [52] Multicenter (Germany, Korea, USA, Netherlands, Canada)	nested case-control	19	76	HL - Hodgkin`s lymphoma, B-cell chronic lymphocytic leukemia/ small lymphocytic lymphoma, Malignant lymphoma nHL - non-Hodgkin`s lymphoma, FL - follicular lymphoma, DLBCL - diffuse large B cell lymphoma, Small B lymphocytic lymphoma, MALT - marginal zone B cell lymphoma	NR	DAS28-4(ESR) for cases - 6.1±1.3, for controls - 6.3±1.1 at baseline and for cases - 3.6±1.0, controls - 3.5±1.1 at the time lymphoma was reported HAQ-DI score for cases - 1.2±0.6, for controls - 1.5±0.7 at baseline and for cases - 0.9±0.6, for controls - 0.8±0.7 at the time lymphoma was reported	Patients without malignancy from the same analysis population, individually matched for age (at time of lymphoma event) and gender (case to control ratio 1:4)	Median 60 (range 42-76) Vs. Median 60 (range 42-76)	4/15 Vs. 16/60	Yes (age and sex with case to control ratio of 1:4)	3/13 (1-HL; 2-BCL)	NR	biopsy	NR	NR	in situ hybridization; immunohistochemistry
Tsukui D, 2018 [53] Japan	retrospective	20	NR	Polymorphyc LPD, DLBCL - diffuse large B cell lymphoma, HL - Hodgkin`s lymphoma, HL-like - Hodgkin-like lymphoma, Peripheral T-cell lymphoma	NR	CRP 2.51 (0.05-22.02) Median (range) Steinblocker stage: II 16%, III 37%, IV 47%	NO CONTROL GROUP	Median 70.5 (range 52-84)	1/19	NR	13/20 EBER 4/20 EBV dna	NR	biopsy	EBER	NR	in situ hybridization; PCR DNA
Tokuhira M, 2018 [54] Japan	NR	59	NR	EBV+ DLBCL - EBV+ diffuse large B cell lymphoma, DLBCL-NOS - diffuse large B cell lymphoma not otherwise specified, HL - Hodgkin`s lymphoma, LPD - lymphoproliferative disorder, FL - follicular lymphoma, PTCL-NOS - peripheral T cell lymphoma not otherwise specified, MALT - marginal zone lymphoma of mucosal associated lymphoid tissue, PMLBCL - primary mediastinal large B cell lymphoma, Plasmablastic, Other LPDs	I, II, III, IV	NR	NO CONTROL GROUP	Mean 65.8 (range 39-88)	21/41	NR	Total 27/55 14/25 DLBCL 8/10 HL 4/7 LPD 0/4 FL 0/4 PTCL-NOS 0/2 MALT 0/1 PMLBCL 0/1 Plasmablastic 3/5 others LPDs	NR	biopsy	EBER	NR	in situ hybridization
Carreras J, 2018 [55] Japan	NR	20	NR	DLBCL - diffuse large B cell lymphoma	NR	Low activity in 94% (method not reported) ECOG PS≥2 11% High CRP 58%	NO CONTROL GROUP	Median 70 (range 44-81)	4/16	NR	13/20 (65%)	NR	biopsy	EBER, LMP1, EBNA2	0-7/20 (35%) I-3/20 (15%) II-6/20 (30%) III-4/20 (20%)	in situ hybridization; immunohistochemistry

Daroontum T, 2018 [56] Japan	NR	6	1	EBV+ MCU - EBV+ mucocutaneous ulcer (B cell LPD)	I, II, III, IV	NR	SS - Sjogren syndrome with LPD (EBV+ mucocutaneous ulcer)	75.3 (SD 8.4) Vs. 72 years	1/5 Vs. 0/1	NR	6/6	1/1	biopsy	EBER	NR	in situ hybridization
Gong S, 2018 [57] USA	NR	2	8	Marginal zone lymphoma	NR	NR	Marginal zone lymphoma with history of other diseases than RA	Median 58.5 (range 54-63) Vs. Median 48 (range 18-78)	0/2 Vs. 4/4	NR	2/2	8/8	biopsy	EBER, LMP1, EBNA2	I - 2/2 i 4/6 in controls II- 0/2 i 2/6 in controls	in situ hybridization; immunohistochemistry
Marcelis L, 2018 [58] Belgium	NR	28	47	HL - Hodgkin`s lymphoma, NHL - non-Hodgkin`s lymphoma, Polymorphic B-cell LPD, Non-destructive LPD, Unclassifiable	I, II, III, IV	NR	LPD patients with other underlying diseases (Crohn`s disease, Ulceratice colitis, Juvenile idiopathic arthritis, Sjogren syndrome, Systemic lupus erythematosus, other)	NR	NR	NR	7	NR	biopsy	EBER	NR	in situ hybridization
Saito S, 2018 [59] Japan	retrospective and prospective	35 Total 25 from retrospective study 10 from prospective study	35 Total 25 for retrospective study 10 for prospective study	HL - Hodgkin`s lymphoma, DLBCL - diffuse large B cell lymphoma, FL - follicular lymphoma, lymphomatoid granulomatosis, MALT - mucosa associated lymphoid tissues lymphoma, NK/T cell lymphoma, reactive hyperplasia, LPD with atypical cell proliferation	I, II, III, IV	CRP Median (range) in regressive group 1.48 (0.26-2.88) in persistent group 0.9 (0.11-2.20) in control group 0.08 (0.03-0.47)	RA patients without LPD treated with MTX matched for age, sex, MTX dose, and RA duration (LPD:control = 1:1) RA patients without LPD treated with MTX clinically matched for age, sex, MTX dose, RA duration	Regressive group median 67 (range 58-73) Persistent group median 67 (range 64-71) Vs. Median 67 (range 64-71)	4/31 Vs. 4/31	Yes (age, sex, MTX dose, and RA duration with LPD:Controls=1:1)	20/30 Total 5/10 prospective study		peripheral blood	EBER, EBV DNA viral load (za prospektivnu grupu)	NR	in situ hybridization real-time PCR (prospective group)
Takanashi S, 2018 [60] Japan	NR	18	NR	BCL- B-cell lymphoma, TCL - T-cell lymphoma, HL - Hodgkin`s lymphoma	I, II, III, IV	CRP 4.5 (0.2-28) mean (range)	NO CONTROL GROUP	Median 66 (range 54-79)	2/16	NR	7/9	NR	biopsy	N/A	NR	N/A
Kurita D, 2019 [61] Japan	NR	219	NR	RH - reactive lymphoid hyperplasia, Poly-LPDs - polymorphic LPDs, B/T-cell non-Hodgkin`s lymphoma, DLBCL - diffuse large B cell lymphoma, indolent B-cell lymphoma, PTCL - peripheral T-cell lymphoma, HL - Hodgkin`s lymphoma	I, II, III, IV	CRP high in 131 patients	NO CONTROL GROUP	RH median 63 (range 26-81) Poly-LPD median 68 (range 42-83) DLBCL median 69 (range 39-88) Indolent-BCL median 66 (range 49-78) PTCL median 70 (range 56-85)	55/129	NR	108/193 total 16/29 RH 23/32 Poly-LPD 48/106 DCBCL 20/26 CHL	NR	biopsy	EBER, LMP1, EBNA2	DCBCL: 0 or I - 5/42 II- 27/42 III -10/42 CHL: O or I - 2/18 II-16/18 III -0/18 Poly-LPD: 0 or I - 1/10 II - 8/10 III- 1/10	in situ hybridization, immunohistochemistry

Satou A, 2019a [62] Japan	case series	9	NR	DLBCL - diffuse large B cell lymphoma, EBVMCU - EBV+ mucocutaneous ulcer, HL - Hodgkin`s lymphoma, polymorphic LPD, HL-like LPD - Hodgkin`s like lymphoma, TCL - T-cell lymphoma	NR	DAS28-CRP moderate (2.7-4.1) n=6 low (<2.7) n=1 clinical remission (<2.3) n=2	NO CONTROL GROUP	Median 75 (range 48-87)	4/5	NR	9/9	NR	biopsy	EBER, LMP1, EBNA2	I - 1/8<br>II - 7/8	in situ hybridization, immunohistochemistry
Satou A, 2019b [63] Japan	NR	61	NR	BCL - B-cell lymphoma, TCL - T-cell lymphoma	III/IV	NR	NO CONTROL GROUP	RA-LPD TCL - median 70 (range 56-85) RA-LPD BCL - median 68 (range 47-82)	25/36	NR	Total 21/61 1/28 MTX T cell LPD 20/33 MTX B cell LPD	NR	biopsy	EBER	NR	in situ hybridization
Yoshifuji K, 2019 [64] Japan	NR	6	NR	HL - Hodgkin`s lymphoma	NR	NR	NO CONTROL GROUP	Median 58 (range 48-72)	2/4	NR	5/6	NR	biopsy	EBER	NR	in situ hybridization
Fujimoto K, 2020 [65] Japan	NR	81	NR	DLBCL - diffuse large B cell lymphoma, polymorphic LPD, HL - Hodgkin`s lymphoma, mantle cell lymphoma, lymphoplasmacytic lymphoma, FL - follicular lymphoma, peripheral T-cell lymphoma, enteropathy-associated T-cell lymphoma, anaplastic large cell lymphoma	I, II, III, IV	NR	NO CONTROL GROUP	Median 69 (range 35-87)	26/55	NR	29/67 Total EBER DLBCL 17/45 HL 5/6 polymorphic LPD 6/10 1/6 others 18/24 Total EBV DNA	NR	biopsy	EBER; EBV DNA	NR	in situ hybridization; pcr
Ikeda T, 2020 [66] Japan	NR	29 EBVMCU	5	DLBCL - diffuse large B cell lymphoma, EBVMCU - EBV+ mucocutaneous ulcer	NR	NR	immunosupressed patients of different causes than RA therapy, (age, polycythemia, polymyalgia rheumatica)	73.21 (SD 8.15)<br>Vs.<br>74.2 (SD 4.66)	12/17<br>Vs.<br>4/1	NR	29/29	5/5	biopsy	EBER	NR	in situ hybridization
Kohno K, 2020 [67] Japan	NR	45 Total<br>9 HL<br>15 DLBCL<br>21 poly-B-LPD	NR	HL - Hodgkin`s lymphoma, DLBCL - diffuse large B cell lymphoma, Poly-B-LPD - polymoprhic B-cell LPD, Other	I, II, III, IV	NR	NO CONTROL GROUP	HL median 68 (range 48-82) DLBCL - median 68 (range 53-82) Poly-B-LPD - median 68 (range 39-80)	17/28	NR	30/45 Total 8/9 HL 6/15 DLBCL 16/21 poly-B-LPD	NR	biopsy	EBER	I – 2<br>II – 16<br>III - 2	in situ hybridization

Laurent C, 2020 [68] Multicenter (France, USA, UK, Germany)	NR	2	12 Total 7 none 3 other LPDs 2 other diseases	HL - Hodgkin`s lymphoma	NR	NR	- LPD (Hodgkin`s lymphoma) patients without history of any disease - LPD (Hodgkin`s lymphoma) patients with previous other LPDs (FL - follicular lymphoma, DLBCL - diffuse large B cell lymphoma, T-LGL leukemia) - LPD (Hodgkin`s lymphoma) patienyts with previous sarcoidosis, LCH - Langerhans cell histocytosis	42.5 (SD 23.33) Vs. 47.5 (range 13-89)	2/0 Vs. 8/4	NR	1/2	6/11 Total 4/6 none 1/3 other LPDs 1/2 other diseases	biopsy	EBER	NR	in situ hybridization
Matsubayashi S, 2020 [69] Japan	case series	2	1	DLBCL - diffuse large B cell lymphoma	NR	NR	psoriasis and LPD patient treated with MTX	Median 69 (range 63-75) Vs. 42 years	0/2 Vs. 1/0	NR	1/2	1/1	biopsy	EBER	NR	in situ hybridization
Shiraiwa S, 2020 [70] Japan	NR	48	NR	EBVMCL - EBV-mucocutaneous lesions, DLBCL - diffuse large B cell lymphoma, Hodgkin-like lymphoma, HL - Hodgkin`s lymphoma, Other	I, II, III, IV	NR	NO CONTROL GROUP	EBVMCL - gingival swelling median 79 (range 74-83) EBVMCL – EBVMCU median 70 (range 55-90) DLBCL median 69 (range 44-81) HLL median 64 (range 51-70) CHL median 63 (range 41-83) Other median 68 (range 47-83)	12/36	NR	37/48 EBER 28/37 LMP1 7/32 EBNA2	NR	biopsy	EBER; LMP1: EBNA2	I – 4 II – 21 III - 7	in situ hybridization, immunohistochemistry
Tokuhira M, 2020 [71] Japan	NR	33	NR	DLBCL - diffuse large B cell lymphoma, HL -Hodgkin`s lymphoma LPDs	NR	NR	NO CONTROL GROUP	Median 65 (range 36-86)	13/20	NR	9/33	NR	biopsy	EBER	NR	in situ hybridization
Yamada C, 2020 [72] Japan	NR	19	10	HL - Hodgkin`s lymphoma, DLBCL - diffuse large B cell lymphoma, FL - follicular lymphoma, AITL - angioimmunoblstic T cell lymphoma	NR	DAS28-CRP Median (range) 2.67 (1.05-10.7) for all RA 3.63 (2.23-5.75) for RA-RLH 2.59 (1.05-10.7) for RA-LPD	RA-RLH (reactive lymph node hyperplasia)	Median 59 (range 31-72) Vs. Median 68 (range 47-84)	9/10 Vs. 4/6	NR	11/19 EBER 10/11 lmp1 1/11 EBNA2 9/13 EA IgG 2/14 VCA IgM 13/14 VCA IgG 11/12 EBNA IgG	1/10 EBER 0/1 lmp1 0/1 EBNA2 2/3 EA IgG 2/3 VCA IgM 3/3 VCA IgG 3/3 EBNA IgG	biopsy	EA IgG, VCA IgM IgG, EBNA IgG; EBER, LMP1, EBNA2	I - 3/12 II - 9/1 III - 1/11	ELISA, in situ hybridization, immunohistochemistry

Kondo M, 2021 [73] Japan	retrospective observational	12	NR	DLBCL - diffuse large B cell lymphoma, TCL - T cell lymphoma, HL - Hodgkin`s lymphoma, MALT - mucosa-associated lymphoid tissue lymphoma	NR	DAS28-ESR 2.67±0.78 mean for 11 patients (1 not evaluated) CRP 2.26 mean for all 12 patients	NO CONTROL GROUP	68.1 (SD 9.3)	7/5	NR	6/11	NR	biopsy	EBER	NR	in situ hybridization
Kuramoto N, 2021 [74] Japan	NR	216 Total 71 DLBCL 20HL 12 Reactive lymphoid hyperplasia 9 Unclassifiable B cell lymphoma 8 FL 6 MZL 5 Hodgkin-like 4 peripheral T-cell lymphoma 3 lymphomatoid granulomatosis 2 composite lymphoma 2 poly-LPDs 1 lymphoplasmacytic lymphoma 1 extranodal NK/T cell lymphoma	NR	DLBCL - diffuse large B cell lymphoma, HL - Hodgkin`s lymphoma, EBVMCU - EBV+ mucocutaneous ulcer, Reactive lymphoid hyperplasia, Unclassifiable B cell lymphoma, MZL - marginal zone lymphoma, Hodgkin-like lymphoma, Peripheral T-cell lymphoma, Lymphomatoid granulomatosis, Composite lymphoma, Poly-LPDs - polymorphic LPDs, Lymphoplasmacytic lymphoma, Extranodal NK/T cell lymphoma	III/IV	DAS28-CRP Median (range) 2.92 (2.05-3.78) DAS28-ESR 3.52 (2.76-4.80) CDAI 6.0 (1.9-12.5) SDAI 8.4 (3.0-19.1) CRP 4.54 (1.48-5.00)	NO CONTROL GROUP	Median 67 (range 60-73)	50/166	NR	42/136 total	NR	biopsy	EBER	NR	in situ hybridization
Nakano K, 2021 [75] Japan	NR	51	NR	DLBCL - diffuse large B cell lymphoma, HL - Hodgkin`s lymphoma, Poly-LPD - polymorphic LPD, plasmablastic lymphoma, B-cell lymphoma, FL - follicular lymphoma, MALT - mucosa-associated lymphoid tissue lymphoma, reactive lymphadenitis, Other	III/IV	DAS28-ESR median (range) 4.2 (2.9-5.1) in regressive patients 3.6 (3.0-5.6) in persistent patients CRP 2.3 (0.3-4.6) in regressive patients 1.9 (0.7-8.3) in persistent patients	NO CONTROL GROUP	Regressive group - median 66.5 (range 34.7-74.0) Persistent group - median 67 (range 62.5-73.5)	12/39	NR	Total 19/51 7/26 regressive LPDs patients 12/25 persistant LPDs patients	NR	biopsy	EBER	NR	in situ hybridization

Takada H, 2021 [76] Japan	case series	195 Biopsy confirmed LPDs<br>79 DLBCL<br>21 HL<br>15 EBVMCU<br>12 Reactive lymphoid hyperplasia<br>11 Unclassifiable B cell lymphoma<br>10 FL<br>9 MZL<br>5 Hodgkin-like<br>4 peripheral T-cell lymphoma<br>3 lymphomatoid granulomatosis<br>2 angioimmunoblastic T-cell lymphoma<br>2 composite lymphoma<br>2 Poly-LPDs<br>1 Extranodal NK/T cell lymphoma<br>1 lymphoplasmacytic lymphoma<br>18 Other	37	DLBCL - diffuse large B cell lymphoma, HL - Hodgkin`s lymphoma, EBVMCU - EBV+ mucocutaneous ulcer, Reactive lymphoid hyperplasia, Unclassifiable B cell lymphoma, MZL - marginal zone lymphoma, HLL - Hodgkin-like lymphoma, PTCL and AITL - Peripheral T-cell lymphoma, Lymphomatoid granulomatosis, Angioimmunoblastic T-cell lymphoma, Composite lymphoma, Poly-LPDs - polymorphic LPDs, Extranodal NK/T cell lymphoma, Lymphoplasmacytic lymphoma, Others	I, II, III, IV	DAS28-CRP med (IQR)<br>2.92 (2.05-3.78) for all patients<br>3.01 (2.06-3.83) biopsy confirmed LPDs<br>2.85 (1.98-3.25) clinical LPDs	RA patients with clinical LPDs (biopsy not confirmed)	Median 67 (range 60-73)<br>Vs.<br>Median 66 (range 59-72)	43/152<br>Vs.<br>10/27	NR	74/136 Total<br>19/50 DLBCL<br>14/18 CHL<br>15/15 EBVMCU<br>6/10 RLH<br>0/2 FL<br>1/4 MZL<br>1/5 PTCL AITL<br>5/5 HLL	NR	biopsy	EBER	NR	In situ hybridization
Fujimoto M, 2021 [65] Japan	NR	2	11	EBVMCU	NR	NR	EBVMCU with no immunosupression th	over 70 years<br>Vs.<br>over 70 years	NR		2	11	biopsy			NR
He J, 2021 [77] Australia	case series	2	NR	Case 1 - HL - Hodgkin`s lymphoma<br>Case 2 - Lymphomatoid granulomatosis	NR	NR	NO CONTROL GROUP	Case 1 - 74 years<br>Case 2 - 67 years	1/1	NR	2	NR	biopsy	NR	NR	PCR, staining

Abbreviations: WHO=World Health Organization, NR=not reported, LPD=lymphoproliferative disorder, RF=rheumatoid factor, ESR=Erythrocyte sedimentation rate

Table S2. Case reports

Author	Year	RA	Th	EBV	Age	Gender
Barry	2008	Yes	MTX	positiv e	78	M
Abe	2018	Yes	Prednisolone Bucillamine Infliximab MTX	positiv e	62	F
Acero	2006	Yes	MTX Sulfasalazine	positiv e	79	F
Agarwal	2004	Yes	NR	NR	52	F
Agarwal	2008	Yes	MTX	NR	57	F
Andrade	2018	Yes	MTX Hydroxychloroquine Etanercept	positiv e	56	F
Aoshima	2019	Yes	MTX	NR	65	F
Arai	2011	Yes	MTX Etanercept	NR	NR	NR
Baba	2016	Yes	MTX	positiv e	79	F
Baird	2002	Yes	MTX	NR	69	F
Bilen	2008	Yes	DMARDs (MTX) NSAIDs	negativ e	53	F
Blanchart	2014	Yes	MTX	positiv e	64	F
Booth	2018	Yes	NR	positiv e	45	M
Boulemden	2009	Not available, poster, conference				
Chen	2019	Yes	MTX Prednisolone Hydroxychloroquine	positiv e	72	F
Chim	2006	Yes	NSAID MTX	positiv e	66	M
Chung	2020	Yes	MTX	negativ e	65	M
Clarke	2007	Yes	MTX	negativ e	91	F
Claudino	2016	Yes	MTX	positiv e	66	F
Craddock	2019	Yes	MTX	positiv e	84	F
Craythorne	2010	Yes	MTX	positiv e	86	F

Cui	2014	Yes	MTX	not done	55	M
De Oliveira Terroso	2014	Yes	MTX	negative	85	M
Dedecker	2015	Yes	MTX	positive	78	M
Derbyshire	2021	Yes	MTX	positive	69	M
Duarte	2010	Yes	Prednisone Sulindac	positive	77	F
Dunleavy	2017	Yes	NR	positive	85	F
Ebeo	2003	Yes	MTX	positive	54	M
febres-Aldana	2020	Yes	Etanercept Tofacitinib Golumumab	positive	71	F
Ferraccioli	1995	Yes	Sulfasalazine Azathioprine Prednisone MTX Leukovorin CSA	positive	61	M
Flipo	1997	Not available				
Fujimoto	2012	Yes	MTX Preednisolone Bucillamine	positive	36	M
Fujiwara-Kuroda	2020	Yes	MTX	positive	sixties	F
Gomyo	2003	Yes	MTX NSAID Parenteral gold Corticosteroides	positive	70	F
Gono	2004	Yes	NSAID Prednisolone Salazosulphapyridine MTX	negative	59	M
Gorodetskiy	2020	Yes	Gold MTX Leflunomide	positive	62	F
Gru	2019	Yes	MTX Corticosteroides	positive	71	M

Hannequin	1998	Yes	MTX<br>Prednisone<br>Indomethacin	NR	69	F
Hashefi	2000	Yes	NR	NR	63	F
Hashimoto	2018	Yes	MTX	positiv<br>e	67	F
Hashizume	2012	No	Prednisolone<br>MTX	negativ<br>e	62	F
Hasselblom	2004	Yes	Prednisolone	positiv<br>e	70	F
Hatachi	2010	Yes	MTX	positiv<br>e	75	F
Hirose	2002	Yes	MTX	positiv<br>e	53	M
Horie	2015	Yes	MTX	positiv<br>e	60	M
Hyo	2011	Yes	MTX	positiv<br>e	75	M
Ikeda	2012	Yes	MTX<br>Adalimumab	positiv<br>e	71	F
Inaba	2015	Yes	MTX<br>TNF inhibitor<br>(Etanercept, Infliximab,<br>Golimumab)	positiv<br>e	39	F
Inuzuka	2013	Yes	Prednisolone<br>MTX	positiv<br>e	64	F
Ishida	2013	Yes	MTX<br>Infliximab	positiv<br>e	76	F
Ishigaki	2018	Yes	MTX	positiv<br>e	83	F
Kaieda	2020	Not available				
Kaito	2021	Yes	MTX	positiv<br>e	84	F
Kakiuchi	2020	Yes	MTX,<br>Prednisolome<br>TAC	positiv<br>e	73	M
Kameda	2007	Yes	Prednisolone,<br>Sulfasalazine<br>Actarit<br>DMARDs<br>Gold injection<br>MTX	positiv<br>e	71	M
Kamekura	2010	Yes	MTX	positiv<br>e	70	F

Kawahara	2015	Yes	MTX	negative	64	M
Kawano	2012	Yes	MTX	negative	60	M
Kawazoe	2020	Yes	MTX	positive	75	M
Kennedy	2006	Yes	MTX<br>Hydroxychloroquine<br>Celecoxib<br>Folic acid	NR	64	M
Kikuchi	2010	Yes	MTX	positive	69	F
Kikuchi	2018	Yes	MTX	positive	73	F
Kobayashi	2015	Yes	Prednisolone<br>MTX	positive	71	M
Kobayashi	2016	Yes	Prednisolone<br>MTX	positive	78	F
Koji	2016	Yes	MTX	positive	48	F
Kojima	2006	Yes	MTX	negative	68	F
Komatsu	2013	Yes	MTX	positive	80	F
Komatsuda	2008	Yes	MTX<br>Prednisolone	positive	63	F
Ishida	2013	Yes	MTX<br>Infliximab	positive	76	F
Kudoh	2014	Yes	MTX	positive	75	F
Kumar	2008	Yes	MTX	positive	56	F
Kurimoto	2016	Yes	MTX	negative	71	F
Kuroda	2014	Yes	MTX	negative	60	M
Lai	2017	Yes	MTX	positive	62	F
Le Goff	1998	Yes	MTX	positive	NR	NR
Liote	1995	Yes	MTX	positive	57	F
Makino	2015	Yes	MTX	positive	47	F

Makis	2017	Yes	MTX	negativ e	67	F
Maruani	2007	Yes	MTX	positiv e	70	F
Maruyama	2020	Yes	MTX	positiv e	69	F
Matsubayas hi	2019	Yes	MTX	NR	67	F
Matsuda	2018	Yes	MTX	positiv e	76	F
Matsumoto	2001	Yes	MTX	positiv e	63	M
Matsumoto	2014	Yes	MTX	negativ e	62	M
Matsuzaki	2018	Yes	Prednisolone MTX	positiv e	80	F
McKelvie	2016	Yes	MTX	positiv e	76	F
Migita	2013	Yes	Bucilamine Prednisolone MTX	positiv e	53	F
Miyashita	2015	Yes	MTX	positiv e	73	F
Miyaza	2019	Yes	MTX	positiv e	68	F
Mizuguchi	2019	Yes	MTX	negativ e	70	M
Mizusawa	2019	Yes	MTX	negativ e	70	F
Mo	2011	Yes	Prednisolone Penicillamine Sulfosalazine MTX infliximab	negativ e	62	M
Moseley	2000	Yes	MTX	NR	61	F
Murayama	2018	Yes	MTX Prednisolone	positiv e	76	F
Nader	2014	Yes	MTX Plaquenil	positiv e	69	M
Nagai	2008	Yes	MTX	positiv e	77	F
Nagata	2015	Yes	MTX	positiv e	39	F

Naidu	2014	Yes	MTX Prednisolone	positive	81	F
Nakanishi	2011	Yes	MTX	positive	72	F
Namiki	2018	Yes	MTX Prednisolone	positive	64	F
Nemoto	2010	Yes	MTX	positive	60	M
Niewold	2003	Yes	MTX	NR	78	F
Niimi	2019	Yes	MTX	negative	63	F
Nishida	2019	Yes	MTX	positive	73	F
Nomura	2021	Yes	MTX	positive	81	F
Obata	2020	Yes	MTX	positive	58	F
Ochi	2013	Yes	MTX	positive	76	F
Oda	2015	Yes	Adalimumab MTX	positive	24	F
Oebisu	2018	Yes	MTX Etanercept	positive	46	F
Ogasawa	2007	Yes	MTX Prednisolone	positive	71	M
Ohkura	2015	Yes	MTX	positive	70	M
Oiwa	2014	Yes	MTX Prednisolone Folic acid Diclofenac sodium	positive	65	F
Oka	2018	Yes	MTX	positive	85	F
Omori	2021	Yes	MTX Prednisolone	positive	71	M
Park	2008	Yes	Etanercept Tofacitinib Golumumab	positive	64	F
Parker	2008	Yes	MTX Infliximab	positive	60	F
Pastor-Nieto	2009	Yes	MTX	positive	79	F
Pfistershammer	2010	Yes	MTX	negative	67	F

Prades	2003	Yes	MTX	NR	52	M
Raja	2010	Yes	Prednisone	NR	40	M
Ramos-Baena	2021	Yes	MTX	positive	53	F
Ravi	2018	Yes	MTX	negative	59	F
Saburi	2018	Yes	Steroids<br>MTX<br>Infliximab	positive	59	M
Sadasivam	2014	Yes	MTX	positive	65	M
Sakaguchi	2019	Yes	MTX<br>Tocilizumab	positive	44	F
Sakurai	2013	Not available				
Saleh	2016	Yes	MTX	positive	66	F
Satoh	2009	Yes	MTX	positive	73	F
Sekiguchi	2015	Yes	Salazosulfapyridine<br>MTX	positive	61	F
Shimada	2015	Yes	MTX<br>Adalimumab	positive	between 60 and 70	F
Shimizu	2017	Yes	MTX<br>Prednisolone	negative	74	M
Shimura	2009	Yes	Corticosteroids<br>MTX	positive	80	F
Shinoda	2014	Yes	MTX	positive	71	M
Shoda	2006	Yes	MTX	positive	71	F
Soubrier	2006	Yes	MTX	positive	48	F
Stewart	2001	Yes	MTX	positive	55	M
Suemori	2015	Yes	MTX<br>Prednisolone<br>Sulfasalazine	positive	79	F
Sugiyama	2021	Yes	MTX<br>Prednisolone	positive	66	F
Suzuki	2018	Yes	MTX	positive	61	F
Svensson	2006	Yes	MTX	negative	48	F

Tajima	2015	Yes	MTX	positiv e	64	F
Takahashi	2014	Yes	MTX	positiv e	70	F
Takajo	2018	Yes	MTX	negativ e	74	F
Takei	2017	Yes	MTX	positiv e	65	F
Takemori	2012	Yes	MTX Salazosulfapyridine Bucillamine Prednisolone NSAIDs	positiv e	60	F
Tamura	2016	Not available				
Tanaka	2020	Yes	MTX	positiv e	68	F
Tatsumi	2014	Yes	MTX	positiv e	67	F
Terroso	2014	Yes	MTX	negativ e	85	NR
Thomason	1996	Yes	MTX Prednisone	positiv e	60	F
Tokuhira	2017	Yes	NSAIDs DMARDs	positiv e	60	F
Tokuyama	2014	Yes	MTX	positiv e	68	F
Toyama	2019	Yes	MTX Tacrolimus	positiv e	71	F
Toyonaga	2019	Yes	MTX	negativ e	70	F
Tsukazaki	2017	Yes	Infliximab MTX	positiv e	88	F
Tsunemine	2019	Yes	MTX	negativ e	69	F
Tsuruoka	2020	Yes	MTX	positiv e	70	F
Ujino	2019	Yes	MTX	positiv e	70	F
Uneda	2020	Yes	MTX	positiv e	68	F
Uneda	2008	Yes	MTX	positiv e	70	F
Van Haarlem	2000	Yes	MTX	positiv e	59	M

Vassilopoulos	1998	Not available				
Vincent	2002	Yes	MTX	positive	NR	NR
Wang	2012	Yes	MTX	negative	65	F
Weir 3rd	1989	Yes	NR	positive	NR	NR
Wernecke	2021	Yes	MTX	positive	80	F
Yamakawa	2016	Yes	MTX	positive	78	M
Yamakawa	2014	Yes	MTX	NR	70	F
Yokose	1998	Yes	NR	negative	55	F
Yordanova	2019	Yes	MTX	positive	69	M
Yu	2015	Yes	Prednisone Azathioprine	positive	86	F
Zijlmans	1992	Yes	Antimalarial drugs Gold Cyclosporine	positive	58	M

References

1. Kamel, O.W.; Van De Rijn, M.; Lebrun, D.P.; Weiss, L.M.; Warnke, R.A.; Dorfman, R.F. Lymphoid Neoplasms in Patients With Rheumatoid Arthritis and Dermatomyositis: Frequency of Epstein-Barr Virus and Other Features Associated With lmmunosuppression. *Hum Pathol.* **1994**, *25*, 638–643.

2. Bachman, T.R.; Sawitzke, A.D.; Perkins, S.L.; Ward, J.H.; Cannon, G.W. Methotrexate-associated lymphoma in patients with rheumatoid arthritis: report of two cases. *ARTHRITIS Rheum.* **1996**, *39*, 325–329.

3. A N De Rijn, M. V; Cleary, M.L.; Variakojis, D.; Warnke, R.A.; Chang, P.P.; Kamel, O.W.; van de Rijn, M.; Wamke, R.A. Epstein-Barr virus clonality in lymphomas occurring in patients with rheumatoid arthritis. *ARTHRITIS Rheum.* **1996**, *39*, 638–642.

4. Georgescu, L.; Quinn, G.C.; Schwartzman, S.; Paget, S.A. Lymphoma in Patients With Rheumatoid Arthritis: Association With the Disease State or Methotrexate Treatment. *Semin Arthritis Rheum.* **1997**, *6*, 794–804.

5. Natkunam, Y.; Elenitoba-Johnson, K.S.; Kingma, D.W.; Kamel, O.W. Epstein-Barr virus strain type and latent membrane protein 1 gene deletions in lymphomas in patients with rheumatic diseases. *ARTHRITIS Rheum.* **1997**, *40*, 1152–1156.

6. Kleiman KS, M.M. Methotrexate and lymphoma a presentation of four cases and review of the literature. *J Clin Rheumatol.* **1998**, *4*, 254–259.

7. Kojima, M.; Nakamura, S.; Shimizu, K.; Itoh, H.; Yoshida, K.; Hosomura, Y.; Yamane, N.; Ban, S.; Joshita, T.; Suchi, T. Florid reactive follicular hyperplasia in elderly patients. A clinicopathological study of 23 cases. *Pathol. Res. Pract.* **1998**, *194*, 391–397.

8. Chevrel, G.; Berger, O.; Miossec, P.; Blanc, M.; Jeanneret, J.; Felman, C.; Tebib, J. Hodgkin's disease and B cell lymphoproliferation in rheumatoid arthritis patients treated with methotrexate: a kinetic study of lymph node changes. *ARTHRITIS Rheum.* **1999**, *42*, 1773–1776.

9. Goodlad, J.R.; Hollowood, K.; Smith, M.A.; C Chan, J.K.; M Fletcher, C.D.; R, G.J.; A, S.M.; K C, C.J.; D M, F.C. Primary juxtaarticular soft tissue lymphoma arising in the vicinity of inflamed joints in patients with rheumatoid arthritis. *Histopathology.* **1999**, *34*, 199–204.

10. Menke, D.M.; Griesser, H.; Moder, K.G.; Tefferi, A.; Luthra, H.S.; Cohen, M.D.; Colon-Otero, G.; Lloyd, R. V Lymphomas in Patients With Connective Tissue Disease Comparison of p53 Protein Expression and Latent EBV Infection in Patients Immunosuppressed and Not Immunosuppressed With Methotrexate. *Am J Clin Pathol* **2000**, *113*, 212–218.

11. Dawson TM, Starkebaum G, Wood BL, Willkens RF, G.A. Epstein-Barr virus, methotrexate, and lymphoma in patients with rheumatoid arthritis and primary Sjögren's syndrome: case series. *J Rheumatol* **2001**, *28*, 47–53.

12. Tournadre, A.; D'incan, M.; Dubost, J.J.; Franck, F.; Déchelotte, P.; Souteyrand, P.; Soubrier, M. Cutaneous lymphoma associated with Epstein-Barr virus infection in 2 patients treated with methotrexate. *Mayo Clin. Proc.* **2001**, *76*, 845–848.

13. Mariette, X.; Cazals-Hatem, D.; Warszawki, J.; Liote, R.; Balandraud, N.; Sibilia, J. Lymphomas in rheumatoid arthritis patients treated with methotrexate: a 3-year prospective study in France. *Blood.* **2002**, *99*, 3909–3915.

14. Baecklund, E.; Sundström, C.; Ekbom, A.; Catrina, A.I.; Biberfeld, P.; Feltelius, N.; Klareskog, L. Lymphoma subtypes in patients with rheumatoid arthritis: Increased proportion of diffuse large B cell lymphoma. *Arthritis Rheum.* **2003**, *48*, 1543–1550.

15. Magro, C.M.; Porcu, P.; Ahmad, N.; Klinger, D.; Crowson, A.N.; Nuovo, G. Cutaneous Immunocytoma A Clinical, Histologic, and Phenotypic Study of 11 Cases. *Appl Immunohistochem Mol Morphol.* **2004**, *12*, 216–224.

16. Verma S, Frambach GE, Seilstad KH, Nuovo G, Porcu P, M.C. Epstein-Barr virus-associated B-cell lymphoma in the setting of iatrogenic immune dysregulation presenting initially in the skin. *J Cutan Pathol* **2005**, *32*, 474–83.

17. Au, W.Y.; Ma, E.S.K.; Choy, C.; Chung, L.P.; Fung, T.K.; Liang, R.; Kwong, Y.L. Therapy-related lymphomas in patients with autoimmune diseases after treatment with disease-modifying anti-rheumatic drugs. *Am. J. Hematol.* **2006**, *81*, 5–11.

18. Baecklund, E.; Iliadou, A.; Askling, J.; Ekbom, A.; Backlin, C.; Granath, F.; Catrina, A.I.; Rosenquist, R.; Feltelius, N.; Sundström, C.; et al. Association of chronic inflammation, not its treatment, with increased lymphoma risk in rheumatoid arthritis. *Arthritis Rheum.* **2006**, *54*, 692–701.

19. Xu, J.X.; Hoshida, Y.; Hongyo, T.; Sasaki, T.; Miyazato, H.; Tomita, Y.; Aozasa, K. Analysis of p53 and Bak gene mutations in lymphoproliferative disorders developing in rheumatoid arthritis. *J. Cancer Res. Clin. Oncol.* **2007**, *133*, 125–133.

20. Kojima, M.; Itoh, H.; Hirabayashi, K.; Igarashi, S.; Tamaki, Y.; Murayama, K.; Ogura, H.; Saitoh, R.; Kashiwabara, K.; Takimoto, J.; et al. Methtrexate-associated lymphoproliferative disorders. A clinicopathological study of 13 Japanese cases. *Pathol. Res. Pract.* **2006**, *202*, 679–685.

21. Kojima, M.; Itoh, H.; Shimizu, K.; Saruki, N.; Murayama, K.; Higuchi, K.; Tamaki, Y.; Matsumoto, M.; Hirabayashi, K.; Igarishi, S.; et al. Malignant Lymphoma in Patients with Systemic Rheumatic Disease (Rheumatoid Arthritis, Systemic Lupus Erythematosus, Systemic Sclerosis, and Dermatomyositis): A Clinicopathologic Study of 24 Japanese Cases. *Int. J. Surg. Pathol.* **2006**, *14*, 43–48.

22. Miyazaki, T.; Fujimaki, K.; Shirasugi, Y.; Yoshiba, F.; Ohsaka, M.; Miyazaki, K.; Yamazaki, E.; Sakai, R.; Tamaru, J.I.; Kishi, K.; et al. Remission of lymphoma after withdrawal of methotrexate in rheumatoid arthritis: Relationship with type of latent Epstein-Barr virus infection. *Am. J. Hematol.* **2007**, *82*, 1106–1109.

23. Kleinschmidt-Demasters, B.K.; Damek, D.M.; Lillehei, K.O.; Dogan, A.; Giannini, C. Epstein Barr Virus-Associated Primary CNS Lymphomas in Elderly Patients on Immunosuppressive Medications. *J Neuropathol Exp Neurol.* **2008**, *67*, 1103–1111.

24. Hasserjian, R.P.; Chen, S.; Perkins, S.L.; De Leval, L.; Kinney, M.C.; Barry, T.S.; Said, J.; Lim, M.S.; Finn, W.G.; Medeiros, L.J.; et al. Immunomodulator agent-related lymphoproliferative disorders. *Mod. Pathol.* **2009**, *22*, 1532–1540.

25. Hellgren, K.; Iliadou, A.; Rosenquist, R.; Feltelius, N.; Backlin, C.; Enblad, G.; Askling, J.; Baecklund, E. Rheumatoid arthritis, treatment with corticosteroids and risk of malignant lymphomas: Results from a case-control study. *Ann. Rheum. Dis.* **2010**, *69*, 654–659.

26. Mariette, X.; Tubach, F.; Bagheri, H.; Bardet, M.; Berthelot, J.M.; Gaudin, P.; Heresbach, D.; Martin, A.; Schaeverbeke, T.; Salmon, D.; et al. Lymphoma in patients treated with anti-TNF: Results of the 3-year prospective French RATIO registry. *Ann. Rheum. Dis.* **2010**, *69*, 400–408.

27. Kojima, M.; Nakamura, N.; Tsukamoto, N.; Itoh, H.; Matsuda, H.; Kobayashi, S.; Ueki, K.; Irisawa, H.; Murayama, K.; Igarashi, T.; et al. Atypical Lymphoplasmacytic and Immunoblastic Proliferation of Autoimmune Disease : Clinicopathologic and Immunohistochemical Study of 9 Cases. *J Clin Exp Hematop.* **2010**, *50*, 113–119.

28. Nakamichi, N.; Wada, N.; Kohara, M.; Fukuhara, S.; Sugiyama, H.; Ogawa, H.; Hino, M.; Kanamaru, A.; Kanakura, Y.; Morii, E.; et al. Polymorphous lymphoproliferative disorder: A clinicopathological analysis. *Virchows Arch.* **2010**, *456*, 269–276.

29. Niitsu, N.; Okamoto, M.; Nakamine, H.; Hirano, M. Clinicopathologic correlations of diffuse large B-cell lymphoma in rheumatoid arthritis patients treated with methotrexate. *Cancer Sci.* **2010**, *101*, 1309–1313.

30. Löfström, B.; Backlin, C.; Pettersson, T.; Lundberg, I.E.; Baecklund, E. Expression of APRIL in diffuse large B cell lymphomas from patients with systemic lupus erythematosus and rheumatoid arthritis. *J. Rheumatol.* **2011**, *38*, 1891–1897.

31. Minamimoto, R.; Ito, K.; Kubota, K.; Morooka, M.; Masuda-Miyata, Y.; Hirai, R.; Kitahara, H.; Tanimura, A.; Hagiwara, S.; Dip, G.; et al. Clinical Role of FDG PET/CT for Methotrexate-Related Malignant Lymphoma. *Clin Nucl Med.* **2011**, *36*, 533–537.

32. Tokuhira, M.; Watanabe, R.; Nemoto, T.; Sagawa, M.; Tomikawa, T.; Tamaru, J.I.; Itoyama, S.; Nagasawa, H.; Amano, K.; Kameda, H.; et al. Clinicopathological analyses in patients with other iatrogenic immunodeficiency-associated lymphoproliferative diseases and rheumatoid arthritis. *Leuk. Lymphoma* **2012**, *53*, 616–623.

33. Ichikawa, A.; Arakawa, F.; Kiyasu, J.; Sato, K.; Miyoshi, H.; Niino, D.; Kimura, Y.; Takeuchi, M.; Yoshida, M.; Ishibashi, Y.; et al. Methotrexate/iatrogenic lymphoproliferative disorders in rheumatoid arthritis: Histology, Epstein-Barr virus, and clonality are important predictors of disease progression and regression. *Eur. J. Haematol.* **2013**, *91*, 20–28.

34. Kondo, S.; Tanimoto, K.; Yamada, K.; Yoshimoto, G.; Suematsu, E.; Fujisaki, T.; Oshiro, Y.; Tamura, K.; Takeshita, M.; Okamura, S. Mature T/NK-cell lymphoproliferative disease and Epstein-Barr virus infection are more frequent in patients with rheumatoid arthritis treated with methotrexate. *Virchows Arch.* **2013**, *462*, 399–407.

35. Loo, E.Y.; Medeiros, L.J.; Aladily, T.N.; Hoehn, D.; Kanagal-Shamanna, R.; Young, K.H.; Lin, P.; Bueso-Ramos, C.E.; Manning, J.T.; Patel, K.; et al. Classical Hodgkin Lymphoma Arising in the Setting of Iatrogenic Immunodeficiency A Clinicopathologic Study of 10 Cases. *Am J Surg Pathol.* **2013**, *37*, 1290–1297.

36. Yamakawa, N.; Fujimoto, M.; Kawabata, D.; Terao, C.; Nishikori, M.; Nakashima, R.; Imura, Y.; Yukawa, N.; Yoshifuji, H.; Ohmura, K.; et al. A clinical, pathological, and genetic characterization

of methotrexate-associated lymphoproliferative disorders. *J. Rheumatol.* **2014**, *41*, 293–299.

37. Kameda, T.; Dobashi, H.; Miyatake, N.; Inoo, M.; Onishi, I.; Kurata, N.; Mitsunaka, H.; Kawakami, K.; Fukumoto, T.; Susaki, K.; et al. Association of higher methotrexate dose with lymphoproliferative disease onset in rheumatoid arthritis patients. *Arthritis Care Res.* **2014**, *66*, 1302–1309.

38. Koens, L.; Senff, N.J.; Vermeer, M.H.; Willemze, R.; Jansen, P.M. Methotrexate-associated B-cell Lymphoproliferative Disorders Presenting in the Skin A Clinicopathologic and Immunophenotypical Study of 10 Cases. *Am J Surg Pathol.* **2014**, *38*, 999–1006.

39. Berghen, N.; Teuwen, L.A.; Westhovens, R.; Verschueren, P. Malignancies and anti-TNF therapy in rheumatoid arthritis: a single-center observational cohort study. *Clin. Rheumatol.* **2015**, *34*, 1687–1695.

40. Hashimoto, K.; Nagao, T.; Saito, T.; Kinoshita, H. Methotrexate-associated lymphoproliferative disorders of the tongue developing in patients with rheumatoid arthritis: A report of 2 cases and a review. *Oral Surg. Oral Med. Oral Pathol. Oral Radiol.* **2015**, *119*, e1–e5.

41. Hollander, P.; Rostgaard, K.; Smedby, K.E.; Chang, E.T.; Amini, R.M.; De Nully Brown, P.; Glimelius, B.; Adami, H.O.; Melbye, M.; Glimelius, I.; et al. Autoimmune and atopic disorders and risk of classical hodgkin lymphoma. *Am. J. Epidemiol.* **2015**, *182*, 624–632, doi:10.1093/aje/kwv081.

42. Inui, Y.; Matsuoka, H.; Yakushijin, K.; Okamura, A.; Shimada, T.; Yano, S.; Takeuchi, M.; Ito, M.; Murayama, T.; Yamamoto, K.; et al. Methotrexate-associated lymphoproliferative disorders: Management by watchful waiting and observation of early lymphocyte recovery after methotrexate withdrawal. *Leuk. Lymphoma* **2015**, *56*, 3045–3051.

43. Shimizu, S.; Inokuma, D.; Murata, J.; Kikuchi, K.; Ito, T.; Fukasawa, Y.; Mukai, M.; Moriuchi, R. Cutaneous manifestations of methotrexate-associated lymphoproliferative disorders: Report of two cases and a review of the literature. *Acta Derm. Venereol.* **2015**, *95*, 366–367.

44. Yamada, K.; Oshiro, Y.; Okamura, S.; Fujisaki, T.; Kondo, S.; Nakayama, Y.; Suematsu, E.; Tamura, K.; Takeshita, M. Clinicopathological characteristics and rituximab addition to cytotoxic therapies in patients with rheumatoid arthritis and methotrexate-associated large B lymphoproliferative disorders. *Histopathology* **2015**, *67*, 70–80.

45. Jeon, Y.W.; Yoon, J.H.; Lee, S.E.; Eom, K.S.; Kim, Y.J.; Kim, H.J.; Lee, S.; Min, C.K.; Lee, J.W.; Min, W.S.; et al. Clinical manifestations of autoimmune disease-related non-Hodgkin lymphoma: A Korean single-center, retrospective clinical study. *Korean J. Intern. Med.* **2016**, *31*, 944–952.

46. Watanabe, S.; Manabe, O.; Hirata, K.; Oyama-Manabe, N.; Hattori, N.; Kikuchi, Y.; Kobayashi, K.; Toyonaga, T.; Tamaki, N. The usefulness of 18F-FDG PET/CT for assessing methotrexate-associated lymphoproliferative disorder (MTX-LPD). *BMC Cancer* **2016**, *16*, 635.

47. Carroll, M.; Biswas, N.; Wheller, L.; Shepherd, V.; Strutton, G.M. Uncommon cutaneous lymphoproliferative disorders in two patients with rheumatoid arthritis. *Australas. J. Dermatol.* **2017**, *58*, e101–e104.

48. Ejima-Yamada, K.; Oshiro, Y.; Okamura, S.; Fujisaki, T.; Mihashi, Y.; Tamura, K.; Fukushige, T.; Kojima, M.; Shibuya, K.; Takeshita, M. Epstein–Barr virus infection and gene promoter

hypermethylation in rheumatoid arthritis patients with methotrexate-associated B cell lymphoproliferative disorders. *Virchows Arch.* **2017**, *470*, 205–215.

49. Katsuyama, T.; Sada, K.E.; Yan, M.; Zeggar, S.; Hiramatsu, S.; Miyawaki, Y.; Ohashi, K.; Morishita, M.; Watanabe, H.; Katsuyama, E.; et al. Prognostic factors of methotrexate-associated lymphoproliferative disorders associated with rheumatoid arthritis and plausible application of biological agents. *Mod. Rheumatol.* **2017**, *27*, 773–777.

50. Gion, Y.; Iwaki, N.; Takata, K.; Takeuchi, M.; Nishida, K.; Orita, Y.; Tachibana, T.; Yoshino, T.; Sato, Y. Clinicopathological analysis of methotrexate-associated lymphoproliferative disorders: Comparison of diffuse large B-cell lymphoma and classical Hodgkin lymphoma types. *Cancer Sci.* **2017**, *108*, 1271–1280.

51. Takanashi, S.; Aisa, Y.; Ito, C.; Arakaki, H.; Osada, Y.; Amano, Y.; Hirano, M.; Nakazato, T. Clinical characteristics of methotrexate-associated lymphoproliferative disorders: relationship between absolute lymphocyte count recovery and spontaneous regression. *Rheumatol. Int.* **2017**, *37*, 1629–1633.

52. Mariette, X.; Chen, C.; Biswas, P.; Kwok, K.; Boy, M.G. Lymphoma in the Tofacitinib Rheumatoid Arthritis Clinical Development Program. *Arthritis Care Res.* **2018**, *70*, 685–694.

53. Tsukui, D.; Kanda, H.; Shinozaki-Ushiku, A.; Tateishi, S.; Takeshima, Y.; Nagafuchi, Y.; Sasaki, O.; Iwasaki, Y.; Harada, H.; Shibuya, M.; et al. Polymorphic lymphoproliferative disorders in patients with rheumatoid arthritis are associated with a better clinical outcome. *Mod. Rheumatol.* **2018**, *28*, 621–625.

54. Tokuhira, M.; Saito, S.; Okuyama, A.; Suzuki, K.; Higashi, M.; Momose, S.; Shimizu, T.; Mori, T.; Anan-Nemoto, T.; Amano, K.; et al. Clinicopathologic investigation of methotrexate-induced lymphoproliferative disorders, with a focus on regression. *Leuk. Lymphoma* **2018**, *59*, 1143–1152.

55. Carreras, J.; Kikuti, Y.Y.; Miyaoka, M.; Hiraiwa, S.; Tomita, S.; Ikoma, H.; Kondo, Y.; Shiraiwa, S.; Ando, K.; Sato, S.; et al. Genomic Profile and Pathologic Features of Diffuse Large B-Cell Lymphoma Subtype of Methotrexate-associated Lymphoproliferative Disorder in Rheumatoid Arthritis Patients. *Am J Surg Pathol.* **2018**, *42*, 936–950.

56. Daroontum, T.; Kohno, K.; Eladl, A.E.; Satou, A.; Sakakibara, A.; Matsukage, S.; Yakushiji, N.; Ya-In, C.; Nakamura, S.; Asano, N.; et al. Comparison of Epstein–Barr virus-positive mucocutaneous ulcer associated with treated lymphoma or methotrexate in Japan. *Histopathology* **2018**, *72*, 1115–1127.

57. Gong, S.; Crane, G.M.; Mccall, C.M.; Xiao, W.; Ganapathi, K.A.; Cuka, N.; Davies-Hill, T.; Xi, L.; Raffeld, M.; Pittaluga, S.; et al. Expanding the Spectrum of EBV-positive Marginal Zone Lymphomas A Lesion Associated With Diverse Immunodeficiency Settings. *Am J Surg Pathol.* **2018**, *42*, 1306–1316.

58. Marcelis, L.; Berghen, C.; De Zutter, A.; Biesemans, P.; Vandenberghe, P.; Verhoef, G.; Gheysens, O.; Sagaert, X.; Dierickx, D.; Tousseyn, T. Other immunomodulatory agent-related lymphoproliferative diseases: a single-center series of 72 biopsy-confirmed cases. *Mod. Pathol.* **2018**, *31*, 1457–1469.

59. Saito, S.; Suzuki, K.; Yoshimoto, K.; Kaneko, Y.; Yamaoka, K.; Shimizu, T.; Mori, T.; Okamoto, S.;

Kameyama, K.; Amano, K.; et al. Restoration of decreased T helper 1 and CD8+ T cell subsets is associated with regression of lymphoproliferative disorders developed during methotrexate treatment. *Front. Immunol.* **2018**, *9*, 621.

60. Takanashi, S.; Nakazato, T.; Aisa, Y.; Ito, C.; Arakaki, H.; Osada, Y.; Hirano, M.; Mori, T. The prognostic value of positron emission tomography/computed tomography in rheumatoid arthritis patients with methotrexate-associated lymphoproliferative disorders. *Ann. Hematol.* **2018**, *97*, 1611–1618.

61. Kurita, D.; Miyoshi, H.; Ichikawa, A.; Kato, K.; Imaizumi, Y.; Seki, R.; Sato, K.; Sasaki, Y.; Kawamoto, K.; Shimono, J.; et al. Methotrexate-associated Lymphoproliferative Disorders in Patients With Rheumatoid Arthritis Clinicopathologic Features and Prognostic Factors. *Am J Surg Pathol* **2019**, *43*, 869–884.

62. Satou, A.; Banno, S.; Hanamura, I.; Takahashi, E.; Takahara, T.; Nobata, H.; Katsuno, T.; Takami, A.; Ito, Y.; Ueda, R.; et al. EBV-positive mucocutaneous ulcer arising in rheumatoid arthritis patients treated with methotrexate: Single center series of nine cases. *Pathol. Int.* **2019**, *69*, 21–28.

63. Satou, A.; Tabata, T.; Miyoshi, H.; Kohno, K.; Suzuki, Y.; Yamashita, D.; Shimada, K.; Kawasaki, T.; Sato, Y.; Yoshino, T.; et al. Methotrexate-associated lymphoproliferative disorders of T-cell phenotype: clinicopathological analysis of 28 cases. *Mod. Pathol.* **2019**, *32*, 1135–1146.

64. Yoshifuji, K.; Umezawa, Y.; Ichikawa, A.; Watanabe, K.; Miura, O.; Yamamoto, M. Methotrexate-associated classical hodgkin lymphoma shows distinct clinicopathological features but comparable clinical outcomes with sporadic cases. *In Vivo (Brooklyn).* **2019**, *33*, 1599–1604.

65. Fujimoto, K.; Hatanaka, K.C.; Hatanaka, Y.; Kasahara, I.; Yamamoto, S.; Tsuji, T.; Nakata, M.; Takakuwa, Y.; Haseyama, Y.; Oyamada, Y.; et al. Association of Epstein–Barr virus with regression after withdrawal of immunosuppressive drugs and subsequent progression of iatrogenic immunodeficiency-associated lymphoproliferative disorders in patients with autoimmune diseases. *Hematol. Oncol.* **2020**, *38*, 799–807.

66. Ikeda, T.; Gion, Y.; Sakamoto, M.; Tachibana, T.; Nishikori, A.; Nishimura, M.F.; Yoshino, T.; Sato, Y. Clinicopathological analysis of 34 Japanese patients with EBV-positive mucocutaneous ulcer. *Mod. Pathol.* **2020**, *33*, 2437–2448.

67. Kohno, K.; Suzuki, Y.; Elsayed, A.A.; Sakakibara, A.; Takahara, T.; Satou, A.; Kato, S.; Nakamura, S.; Asano, N. Immunohistochemical Assessment of the Diagnostic Utility of PD-L1 (Clone SP142) for Methotrexate-Associated Lymphoproliferative Disorders with an Emphasis of Neoplastic PD-L1 (Clone SP142)-Positive Classic Hodgkin Lymphoma Type. *Am. J. Clin. Pathol.* **2020**, *153*, 571–582.

68. Laurent, C.; Arber, D.A.; Johnston, P.; Fend, F.; Zamo, A.; Attygalle, A.D. Diagnosis of classic Hodgkin lymphoma on bone marrow biopsy. *Histopathology* **2020**, *76*, 934–941.

69. Matsubayashi, S.; Suzuki, M.; Sakamoto, K.; Izumi, S.; Hojo, M.; Sugiyama, H. Three different CT and FDG PET/CT findings of pulmonary involvement in methotrexate-associated lymphoproliferative disease. *Respirol. Case Reports* **2020**, *8*, e00520.

70. Shiraiwa, S.; Kikuti, Y.Y.; Carreras, J.; Hara, R.; Aoyama, Y.; Ogiya, D.; Suzuki, R.; Toyosaki, M.; Ohmachi, K.; Ogawa, Y.; et al. Clinicopathological evaluation of methotrexate-associated

lymphoproliferative disorders with special focus on Epstein-Barr virus-positive mucocutaneous lesions. *J. Clin. Exp. Hematop.* **2020**, *60*, 159–168, doi:10.3960/jslrt.20041.

71. Tokuhira, M.; Tanaka, Y.; Takahashi, Y.; Kimura, Y.; Tomikawa, T.; Anan, T.; Watanabe, J.; Sagawa, M.; Higashi, M.; Momose, S.; et al. The clinical impact of absolute lymphocyte count in peripheral blood among patients with methotrexate - associated lymphoproliferative disorders. *J. Clin. Exp. Hematop.* **2020**, *60*, 41–50.

72. Yamada, C.; Oguro, E.; Tsuji, S.; Kudo-Tanaka, E.; Teshigawara, S.; Ohshima, S.; Hashimoto, J.; Saeki, Y.; Horiuchi, T.; Iizuka, N.; et al. Pathological assessment of the lymph node biopsies for lymphadenopathy in rheumatoid arthritis. *Mod. Rheumatol.* **2020**, *30*, 835–842.

73. Kondo, M.; Murakawa, Y.; Moriyama, M.; Honda, M.; Sugiura, T.; Onoda, K.; Watanabe, Y.; Kakimaru, H. Distinct decrease in peripheral lymphocytes in EBER-positive cases of MTX-LPD. *Mod. Rheumatol.* **2021**, *31*, 88–93.

74. Kuramoto, N.; Saito, S.; Fujii, T.; Kaneko, Y.; Saito, R.; Tanaka, M.; Takada, H.; Nakano, K.; Saito, K.; Sugimoto, N.; et al. Characteristics of rheumatoid arthritis with immunodeficiency-associated lymphoproliferative disorders to regress spontaneously by the withdrawal of methotrexate and their clinical course: A retrospective, multicenter, case–control study. *Mod. Rheumatol.* **2021**, 1–16.

75. Nakano, K.; Saito, K.; Nawata, A.; Hanami, K.; Kubo, S.; Miyagawa, I.; Fujino, Y.; Nakayamada, S.; Tanaka, Y. Clinical aspects in patients with rheumatoid arthritis complicated with lymphoproliferative disorders without regression after methotrexate withdrawal and treatment for arthritis after regression of lymphoproliferative disorders. *Mod. Rheumatol.* **2021**, *31*, 94–100.

76. Takada H, Kaneko Y, Nakano K, Tanaka M, Fujii T, Saito K, Sugimoto N, Sasaki S, Saito S, Saito R, Kuramoto N, Harigai M, S.Y. Clinicopathological characteristics of lymphoproliferative disorders in 232 patients with rheumatoid arthritis in Japan: A retrospective, multicenter, descriptive study. *Mod. Rheumatol.* **2021**, 1–9.

77. He, J.; Williamson, L.; Cai, K.; Wong, P.; Sturgess, A.; Taper, J.; Manolios, N. Epstein-Barr virus - related lymphoma in rheumatoid arthritis: Implications for long-term usage of immunosuppressive drugs and review of the literature. *Intern. Med. J.* **2021**.
